# Planning, implementing and governing systems-based co-creation: the DISCOVER framework

**DOI:** 10.1186/s12961-023-01076-5

**Published:** 2024-01-08

**Authors:** Niamh Smith, Michail Georgiou, Mohammad S. Jalali, Sebastien Chastin

**Affiliations:** 1 School of Health and Life Sciences, Glasgow Caledonian University, Glasgow, G4 0BA UK; 2 School of Social and Political Sciences, University of Glasgow, Glasgow, G12 8RZ UK; 3 MGH Institute for Technology Assessment, Harvard Medical School, Boston, MA USA; 4 Department of Movement and Sports, Ghent University, Watersportlaan 2, 9000 Ghent, Belgium

**Keywords:** Systems mapping, Systems thinking, Methods, Public health, Group model building, Health, Policy formulation, Co-creation

## Abstract

**Background:**

Increasingly, public health faces challenges requiring complex, multifaceted and multi-sectoral responses. This calls for systems-based approaches that facilitate the kind of collective and collaborative thinking and working required to address complexity. While the literature on systems thinking, system dynamics and the associated methodologies is extensive, there remains little clear guidance on how to plan, govern and implement participatory systems approaches within a co-creation process.

**Methods:**

We used a three-step process to develop DISCOVER, a framework for implementing, and governing systems-based co-creation:

- Stage 1: We conducted a literature analysis of key texts to identify well-documented methods and phases for co-creation using a systems approach, as well as areas where gaps existed.
- Stage 2: We looked for the most appropriate methods and approaches to fill the gaps in the knowledge production chain.
- Stage 3: We developed the framework, identifying how the different tools and approaches fit together end-to-end, from sampling and recruiting participants all the way through to responding with an action plan.

**Results:**

We devised DISCOVER to help guide researchers and stakeholders to collectively respond to complex social, health and wider problems. DISCOVER is a strategic research planning and governance framework that provides an actionable, systematic way to conceptualise complex problems and move from evidence to action, using systems approaches and co-creation. In this article, we introduce the eight-step framework and provide an illustrative case study showcasing its potential. The framework integrates complementary approaches and methods from social network analysis, systems thinking and co-creation literature. The eight steps are followed sequentially but can overlap.

**Conclusions:**

DISCOVER increases rigour and transparency in system approaches to tackling complex issues going from planning to action. It is being piloted in environmental health research but may be suitable to address other complex challenges and could be incorporated into research proposals and protocols for future projects.

**Supplementary Information:**

The online version contains supplementary material available at 10.1186/s12961-023-01076-5.

## Introduction

Systems-based approaches have been adopted widely across many disciplines, and it is therefore near impossible to remain informed on the totality of the systems literature; computer scientists, geographers, economists, psychologists, social scientists and increasingly health professionals and epidemiologists talk using systems language. Despite the extensive range of writings on systems in different subject areas, there are shared conceptual foundations. A system can be defined as an interconnected assembly of components, where the presence of components impacts the system’s behaviour, and the system undergoes changes when components are altered or removed [1, 2]. The arrangement of components in a system collectively serves a specific purpose, sometimes outlined by distinct boundaries that set the system apart from its environment. Over time, systems and their environments co-evolve [3]. Systems can comprise numerous subsystems, each with its components that could also function as independent systems.

All systems are to be regarded as an adaptive whole, as entities. To comprehend and make sense of the complexity of a system, systems thinking can encourage abstract thinking, widening the gaze rather than looking at component parts separately. Systems thinking involves understanding how component parts in the system are linked and connected, appreciating the counterintuitive effects within a system, and recognising that changes to a system may result in unintended consequences [1, 2].

Practitioners of systems thinking must remain aware of their personal perspectives and biases, recognising that their viewpoints shape how they understand systems [1]. Additionally, openness and respect for diverse perspectives are vital within a safe environment, as conflicting interests may arise among individuals [4]. Working with groups of people is not always straightforward and collaborative research often entails navigating challenges arising from diverse perspectives, communication hurdles, and coordination intricacies. Systems approaches offer a valuable means to harmonise and integrate divergent viewpoints by illuminating the interconnectedness and interdependencies among various perspectives [5].

Contextualising the system and identifying potential barriers to future change are also crucial aspects of systems thinking, enabling context-sensitive actions [5]. Finally, it is important to note that models and maps of systems are not replicas of the real world; they don’t offer complete explanations for situations. Employing a systems approach can provide a novel perspective for contemplating and understanding complex causal relations, for example in public health, by reframing and recontextualising what is happening and why.

Systems thinking and systems-based approaches are promising tools to address the complexity in public health [6–8]. Several diverse methods can be considered ‘systems-based’. Most of these involve developing a visual representation of the system as this can aid the interpretation of data and any decision-making processes [9]. Reality is complicated, and so diagrams can help communicate specific features of a situation. Causal Loop Diagrams are visual tools used to represent and analyse complex systems, emphasising the causal relationships between variables [10]. These can be developed by individuals or through participatory approaches like Group Model Building (GMB) [11] and Participatory Systems Mapping (PSM) [4]. GMB/PSM can help address the complex and multifaceted nature of many complex public health issues and encourage collaboration and dialogue between academia, industry, the government and the public, which can lead to more innovative and nuanced approaches to public health research [12].

To date, systems approaches have been used to understand a variety of ‘wicked problems’ within public health, including adoption of new medical technologies [13, 14], the effectiveness of health care systems [8, 15], sustainability of public health interventions [16], substance use [17, 18], the determining factors which influence sedentary behaviours [19, 20] and levels of obesity [21–24], neonatal mortality [25], among others. Traditionally, research in public health has heavily relied on experimental designs, following a deductive process to test hypotheses. While these methods have successfully uncovered disease causes and mechanisms, they might fall short in grasping the complexities of broader health contexts [12]. Complex public health issues require holistic and multi-dimensional approaches; they cannot be entirely resolved through traditional linear, analytical approaches.

Our research team studies the complex relationships between urban blue spaces (e.g. rivers, canals and lakes) and health. Urban blue spaces have shown promise as effective public health assets, but how these spaces should be used, managed and revitalised to affect health positively is still largely unknown [26]. We recognised that any changes to blue spaces affect an extensive system of actors, including land owners, waterway management agencies, third sector organisations, government bodies and local people, to name a few. We needed to understand the needs and experiences of a wide range of actors before any recommendations for how these spaces should be used or managed were endorsed. The need to consider the wider system surrounding blue spaces led us to adopt a systems-based lens to the research and explore the myriad of tools and methods which fall under the umbrella of ‘systems thinking’.

The challenge we now face is that the practice of navigating complexity is complicated. Systems thinking and systems mapping literature is vast and growing rapidly. Before approaching any systems-based project, one must spend time trawling methods and materials. Those who would like to use systems approaches do not often have the resources for this preliminary work. The facilitation of PSM and GMB processes are well established [10, 11]. What is lacking is a procedural meta-methodology to guide researcher through the entire process, end-to-end, from sampling and recruiting participants all the way through to reporting of outcomes. The term ‘meta-methodology’ describes a high-level framework which encompasses multiple subprocesses, enabling the successful management of a research project [27]. This paper aims to develop a heuristic meta-methodology, called DISCOVER, for planning, implementing and governing a systems based process. We use ‘heuristic’ to emphasise the practical problem-solving nature of the meta-methodology. The heuristic meta-methodology compliments and build on exiting systems thinking methods, combining them into an end-to-end workflow to address complex problems.

## Methods

### Developing DISCOVER

We used a three stage process to develop DISCOVER:

Stage 1: We conducted a literature analysis of key texts to identify well-documented methods and phases for co-creation using a systems approach, as well as to identify missing steps in the knowledge production chain.

Stage 2: We looked for the most appropriate methods and approaches to fill the gaps in the knowledge production chain.

Stage 3: We developed the framework, identifying how the different tools and approaches fit together end-to-end, from sampling and recruiting participants all the way through to responding with an action plan.

### Stage 1

Firstly, we reviewed key textbooks on participatory systems mapping and systems approaches [10, 11, 28–30]. Our rationale and aim was to create an over-arching framework that combines diverse system methods and necessary key steps into an end-to-end co-creation process. For this we had to study the different steps required in the process and develop guidance about how to sequence the most appropriate and relevant methods. Therefore, we purposefully only reviewed textbooks initially, as these are foundational starting points. Also, as we were specifically looking at systems thinking and mapping methods, textbooks provide comprehensive explanations and overviews of well-documented research practices. On the other hand, methodological journal articles tend to deal with improving detailed methodological aspects too specific for our purpose and articles that apply system thinking tend to lack specific reflections on how methods were chosen and conducted, mainly due to word limits. From there, we scanned reference lists of the textbooks and looked at key journal articles. While reviewing, we used Cochrane’s ‘Population, Intervention, Comparison, and Outcome’ (PICO) to analyse the literature [31]. PICO is a widely recognised tool to help researchers develop clear and focused questions that can guide their search for relevant literature as well as support in the evaluation of this evidence [31]. We asked:

- P: What population is involved in the systems approach/systems mapping? (Population)
- I: How was systems mapping used? (Intervention)
- C: How did the authors discuss the output in relation to the study’s sample population, and did they acknowledge potential variations in output that may arise from using a different group of participants? (Comparison)
- O: What is the output of the systems mapping process, and to what extent should participants be involved in reviewing and verifying the results? Additionally, how engaged were participants throughout the systems mapping process? (Outcome)

### Stage 2

We engaged with literature on sampling in qualitative [32] and quantitative [33] research to construct a model appropriate to make sampling for systems mapping more transparent.

Social network analysis informed DISCOVER as a database can be created to catalogue organisations and stakeholders associated with the issue, followed by a survey to assess their interconnectedness. The resulting network map offers a comprehensive view of the network’s structure, serving as a crucial foundation for the subsequent phases of research.

This stage combined ‘social network analysis’, ‘purposeful sampling’, and ‘stratified sampling’ to Identify participants. Combining these sampling techniques offers several advantages, including increased representativeness, diversity, and a more nuanced understanding of the target population. Social network analysis centrality measures can be used to identify important or influential individuals within a network. Centrality is a measure of the extent to which an organisation is connected to others within a social network. High network centrality indicates that an organisation or individual is well-connected. By using centrality as an indicator for sampling, organisations with the potential for wide reach, influence over other organisations, and high social capital are represented.

Purposeful sampling is a non-probability sampling method commonly used in qualitative research, where the researcher deliberately selects participants based on specific criteria or characteristics related to the research question or topic [34]. All the different sectors relevant to the issue must be considered, and researchers can aim to include at least one person from each relevant sector. Stratified sampling is more commonly used in quantitative research and ensures a representative and diverse sample by selecting participants based on specific criteria [35]. Participants can also be sampled based on the Quadruple Helix Model to gain a sample from academia, industry, government, and civil society [35]. Combining sampling methods may reduce bias in sampling by creating a more representative selection process of participants to form the co-creation group.

We acknowledged that GMB and PSM are already well-established methodologies. Variations on these methods were reviewed. Variable elicitation and then the connecting of these variables are key stages across all methods, and so these were considered essential to our framework. Furthermore, making the maps digestible enough to be ‘usable’ was important as was ensuring they were representative of peoples’ understanding of the system. It was then important that we explored how the maps may evolve and change through different scenario testing techniques. These steps are already largely covered in the existing literature of GMB and PSM, but there are limitations on suggested methods to use for this.

Finally, it was vital that we considered the impact of the project and the best way to move from evidence to action [36]. In the textbooks we reviewed, finalising the map tended to be the final steps of systems mapping processes and there was little guidance on how to use the maps to enact change [10, 11, 28–30].

From the emerging literature, Co-creation was deemed an appropriate approach for developing policy recommendations. Co-creation, which has its roots in the participatory research paradigm and has been traced back to the 1970s (166), represents a promising approach to public health challenges (167). It can enhance the effectiveness and impact of health interventions, particularly for complex public health issues like the obesity epidemic, persistent poverty, and food insecurity, which often pose challenges to resolving, especially for vulnerable populations (168). Additionally, co-creation addresses the growing demand from the public to be actively involved in research, ensuring that health interventions are better aligned with their specific needs and circumstances (167). Such aims align with the ideas presented in systems thinking and systems mapping literature. Therefore, combining these approaches was an appropriate final step in the framework.

### Stage 3

We used the analysis in Stage 2 to develop the framework, identifying how existing systems thinking and mapping tools as well as other complementary approaches could be placed together to form an end-to-end workflow. We then used the framework for an illustrative case study example and use this throughout this paper to contextualise the theory.

### Case study of north Glasgow

Our research has focused on the canals in North Glasgow as a case study. Socioeconomically, North Glasgow is generally considered to be one of the more deprived areas of the city, with higher rates of poverty, unemployment, and poor health outcomes, compared to other areas [37]. Such parts of Glasgow also experience lower-quality greenspace than wealthier neighbourhoods [38]. We have researched the health benefits of living near the canals and found that its regeneration has led to improved health outcomes, including reduced risk of non-communicable diseases [39] and the canal being a protective factor against the negative impact of socio-economic deprivation on mental health [40]. Qualitative research supported these findings with evidence supporting the canals as local therapeutic landscapes [41].

With our evidence to support the benefits of living near blue spaces, we sought to understand how best to use and manage them for population health benefits.

Our DISCOVER process was conducted during the COVID-19 pandemic. This led to our process being conducted entirely remotely using digital communications and mapping tools. Ethical approval was obtained from the School of Health and Life Sciences at Glasgow Caledonian University (code:HLS/PSWAHS/19/208).

## Results

On analysis of the key texbooks, we recognised that there are many tools to guide the facilitation of participatory mapping. For example, Hovmand et al. (2013) introduce group model building using structured small-group facilitated exercises which they call ‘scripts’ [28], and Barbrook-Johnson and Penn (2022) cover many different types of systems mapping, including Participatory Systems Mapping (PSM) [10]. However, what was lacking was the preceding and final stages needed to address complex problems. For example, concerning who to invite as participants for GMB workshops, Scriptapedia’s has a ‘who is in the room?’ section, but does not guide the user on how to select such people [28]. Additionally, Barbrook-Johnson and Penn (2022) suggest that researchers should “cover all parts of the system… keep group size small but maintain diversity” (p69) and “you want to get a good spread of people, representing different views and knowledge of different parts of a system” (p146), but do not explicitly say how this might be achieved in practice [10]. Furthermore, Van den Belt has a large section on ‘setting the participant group’ (p64) for Mediated Modelling workshops, but again this is abstract and theoretical and does not provide a structured approach [30].

A further limitation of existing methodologies is that there is little in the way of actionable output from many participatory systems mapping exercises; the maps are viewed as a final step, but there is little evidence of how to use these maps for action. Therefore, we saw a need for a final stage which aims to respond to the problem.

To encapsulate and simplify the process of planning, implementing and governing a system based co-creation approach, we created an acronym to systemise research and increase the robustness. The word DISCOVER was an effective acronym as following the framework allows users to discover responses to complex problems. DISCOVER is a simplified, practical framework that can be adopted to assist users with governing and conducting their systems-based co-creation process and allow them to arrive at a response to a problem strategically and transparently.

**Fig. 1:**
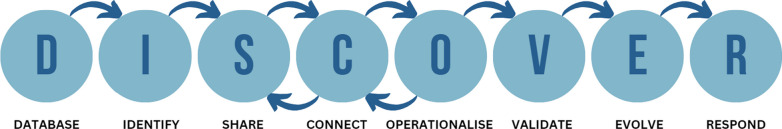
DISCOVER framework for planning, implementing and governing systems-based co-creation. Arrows show direction of travel through the meta-methodology, acknowledging that at some stages the system mapping process is iterative and stages may be revisited

We devised DISCOVER as a strategic, eight-step framework that provides an actionable, systematic way to address complex problems using participatory co-creation. The eight steps are followed sequentially but can overlap (Fig. 1). The process is iterative and previous steps can be revisited as needed as understanding of the problem changes.

The DISCOVER framework enhances existing systems mapping processes by providing a meta-methodology which is systematic and transparent. It firstly incorporates more careful consideration of participant sampling (Database and Identify), and secondly, encourages practical strategies for utilising the system maps to drive positive change (Respond). The steps, aims, objectives and suggested methods for each step are detailed in Table 1.

**Table 1:** Steps of DISCOVER and the associated aims, objectives, suggested methods and references

Step	Aim	Objectives	Suggested methods
D—Create a DATABASE of stakeholders	To systematically map the network of people and organisations operating around the issue	D1. Establish an exhaustive database of stakeholders interested in the problem D2. Collect information about all organisations and how they work together D3. Produce a network map from this database using mapping software	A systematic search of stakeholders, incorporating brainstorming, web mapping searches, networking events, social media, engaging with experts, and using existing contacts [42] Collect socio-metric data through an online survey asking all stakeholders about their network [44] Network mapping and analysis to visualise relationships between people in a network, showing who the actors are, how they are related and how these relationships are characterised [43]
I—IDENTIFY appropriate participants	To identify a representative sample of individuals to participate in systems mapping sessions	I1. Devise a checklist for purposeful sampling I2. Use this checklist to identify and select participants for the co-creation sessions	Use purposeful sampling and stratify by the Quadruple Helix Model [35], social network analysis [44] and the breadth of expertise required to respond to the complex issue [35]
S—Bring identified participants together to SHARE their experiences and expertise	To share knowledge and evidence on the problem	S1. The identified individuals meet within a co-creation session (in person or virtually) facilitated by a researcher S2. Researchers conduct variable elicitation S3. Researchers document the origin of all data so that there is transparency	Identified participants are invited to a participatory mapping session. These can follow Group Model Building scripts [28] and/or Participatory Systems Mapping guides [4]
C—Participants CONNECT their ideas through a systems mapping exercise	To connect ideas and explore relationships between variables	C1. Participants Connect variables	Construct an inter-relationship digraph (IRD) [45–47]. Participants are invited to connect elicited variables as appropriate
O—OPERATIONALISE this map so that it is usable and depicts the full system	To operationalise the system map so that it is actionable	O1. Researchers analyse the IRD and identify drivers and outcomes O2. Researchers operationalise the IRD into a Causal Loop Diagrams using a software package	Researchers dissect the connections and look to refine and operationalise the model to be operational for decision-making [47] Researchers develop Causal Loop Diagrams [48] using packages like Vensim, Stella, KUMU, STICKE, among others
V—VALIDATE the map with those involved in its creation and with other stakeholders	To validate the trustworthiness of the systems map with participants	V1. Member-check the operationalised map with stakeholders to test clarity, ensure an accurate representation of relationships and check for missing information	Researchers member check with participants [25] Use Guba and Lincoln’s framework for ensuring trustworthiness and rigour in qualitative primary data collection, which assesses research on four criteria: credibility, transferability, dependability, and confirmability [49]
E—Bring decision-makers together to test the map and see actions EVOLVE	The map and action ideas evolve through scenario building and stress testing	E1. Researchers identify key leverage points in the system using network analysis and narrative analysis	Leverage points signal areas where we can intervene in a system to bring about positive change. Such engagement can stimulate new forms of action to propose ambitious changes in complex situations [50]
R—RESPOND to the complex problem by collectively developing a set of recommendations	To respond to the problem with an action plan	R1. Co-create an action plan, a shared vision, to respond to the problem R2. Identify common, measurable action points and create pathways for increased future collaboration R3. Use the action plan as evidence to shape policy	Can adapt the ‘Action Ideas’ script from Scriptapedia to discuss potential interventions in the system and areas of action [51] Use co-creation methods with influential stakeholders and decision makers to develop the action plan, based on the evidence from the systems map [52]

### Database

Network mapping and analysis is a way to visualise relationships between people in a network, showing who the actors are, how they are related and how these relationships are characterised [43]. By examining the patterns and structures of relationships, it is possible to gain insights into the dynamics of the network and how it functions as a whole. For example, network mapping can be used to identify central players or hubs within a network and to understand the role different individuals or groups play in the network.

Data for network analysis can be obtained in two ways. Egocentric data can be collected by asking one person to describe their network and who is connected [53]. Socio-metric data is the complete set of relations among people in a network, including both direct and indirect ties and is collected by asking many people about the network [53]. Socio-metric data collection is most appropriate for this step.

### Blue space example

**Fig. 2:**
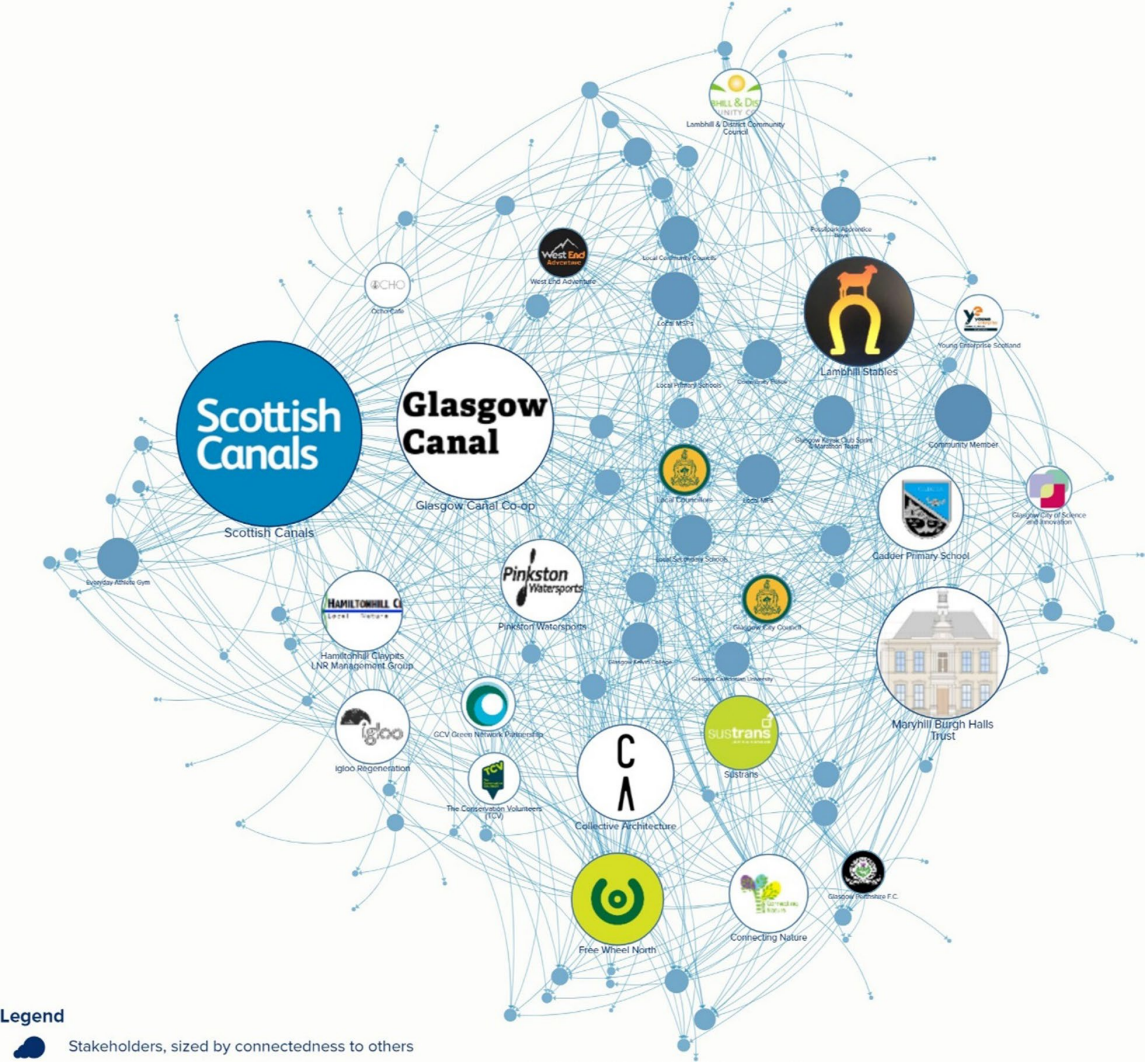
Network map of organisations operating around the canals in North Glasgow

We collated an exhaustive database of all organisations operating around the canals in Glasgow, our study site, through a systematic geographical search of businesses, social enterprises and third-sector organisations. We also used contacts at our university, Google maps, and snowballed contacts from partner organisations. We searched social media and attended community engagement breakfasts in North Glasgow. Socio-metric data were collected using an online survey asking participants to provide a short biography of the organisation they represented. They were asked to rate their collaboration status with other organisations: Yes, we currently collaborate; Yes, we have collaborated in the past; No, but we would like to collaborate in the future; No, and we are not interested in future collaboration. The final part of the survey asked participants if they were interested in the next steps in the research. If interested, they could provide contact details. Finally, participants had the opportunity to suggest other organisations that may have been missed, which allowed for snowball sampling. The research team piloted this survey before it was disseminated. The survey was distributed through email and social media (Facebook and Twitter), with personalised follow-up emails at fortnightly intervals. Data collected from the survey was mapped using KUMU, an online visualisation tool that allows users to create interactive diagrams and network maps (Fig. 2). The social network map was instrumental to establishing the research context; from this map we could identify the most influential, most connected organisations as well as those which are smaller, but may still be significant to our research. The experience helped us become more familiar with the various types of organisations, which in turn made us more knowledgeable of the context, politics and nuances of relationships when conducting the co-creation sessions.

### Identify

This step employed stratified purposeful sampling which ensures a representative and diverse sample by selecting participants based on specific criteria [35]. The ‘Identify’ sample is stratified using the Quadruple Helix Model, sectors of expertise and network analysis metrics. This process reduces bias in sampling by creating a fairer selection process of participants to form the co-creation group.

The Quadruple Helix Model recognises the need for collaboration between science, policy, industry and society within innovation [35]. The breadth and diversity of participants are deemed critical for participatory research over any form of statistical representation [35]. Therefore, deciding on the sectors of expertise required to respond to a problem is essential. Expert groupings should include individuals and organisations who are affected by the changes as well as those who can affect change.

Once the data is mapped, network analysis can be computed to help assist which organisations should be identified as participants. The most influential individuals within a network can be identified using centrality metrics. Centrality is a measure of the extent to which an organisation is connected to others within a social network. High network centrality indicates that an organisation or individual is well-connected, potentially highlighting the organisations’ wide reach, influence over other organisations or their social capital in having capacity to build trust across the network.

The network map can be validated through member checking with several individuals to scrutinise the connections. Furthermore, it may be interesting to note the degree to which different types of organisations stick together (homophily) and the extent to which different organisations reciprocate in acknowledging the collaborative relationship [54]. Homophily is interesting to consider as to understand social structure and the underlying social forces that influence the formation of relationships and can also help explain why certain groups may be disadvantaged in terms of access to resources and opportunities, as they may be more likely to form ties with individuals who are similar to them but also share their lack of resources [55].

### Blue space example

The criteria developed for our project are displayed in Additional file 1: Appendix 1. Researchers exploring methods for co-creation advise 10–12 participants to be included in the co-creation process to allow for dropout throughout the process [56]. Each participant was represented by a number, and collectively, all criteria should be met. This was a flexible and iterative process; if required, people could be invited to participate at a later stage as needed.

### Share

The Share step brings participants together to elicit the variables they understand to be a part of the system to gain a comprehensive understanding of the system as a whole. Hovmand et al. (2012) propose script for group model building [51]. We detail an adapted script used for the ‘Share’ step in Additional file 1: Appendix 2. The facilitator asks participants to note as many variables as possible on post-it notes or the digital equivalent. These are then discussed as a group and duplicates are removed. Where there are conflicting views, the variables can be ‘parked’ for discussion later to avoid disrupting the flow. Systems thinking requires practitioners to acknowledge multiple worldviews; no one person has the same understanding of any situation. Conflicting opinions are valuable and ensure that ideas are challenged [5]. While achieving full consensus may be challenging in many cases, it remains crucial to reach a point where a pragmatic decision can be made. Without reaching this stage, progress and forward momentum can be impeded. Participatory systems mapping involves eliciting core variables from participants and uses scripts to prompt the co-design of a map [57]. Stakeholders may also have varying views and understanding of other issues, including how to define and characterise the complex problem under investigation and the boundaries of the system. Therefore, it is important at the start of the process to establish the researchers’ understanding of the issue and explain the rationale behind the project and research question.

### Blue space example

We ran an online mapping session, as COVID-19 restrictions prevented face-to-face research. Our goal was to have participants elicit all key variables that we should consider when thinking about blue spaces and health. We used Microsoft Teams and a Mural online whiteboard for the Share and Connect steps. Participants added their ideas to the Mural board. It was important that they shared variables based on evidence garnered from research, their lived experiences or professional expertise.

**Fig. 3:**
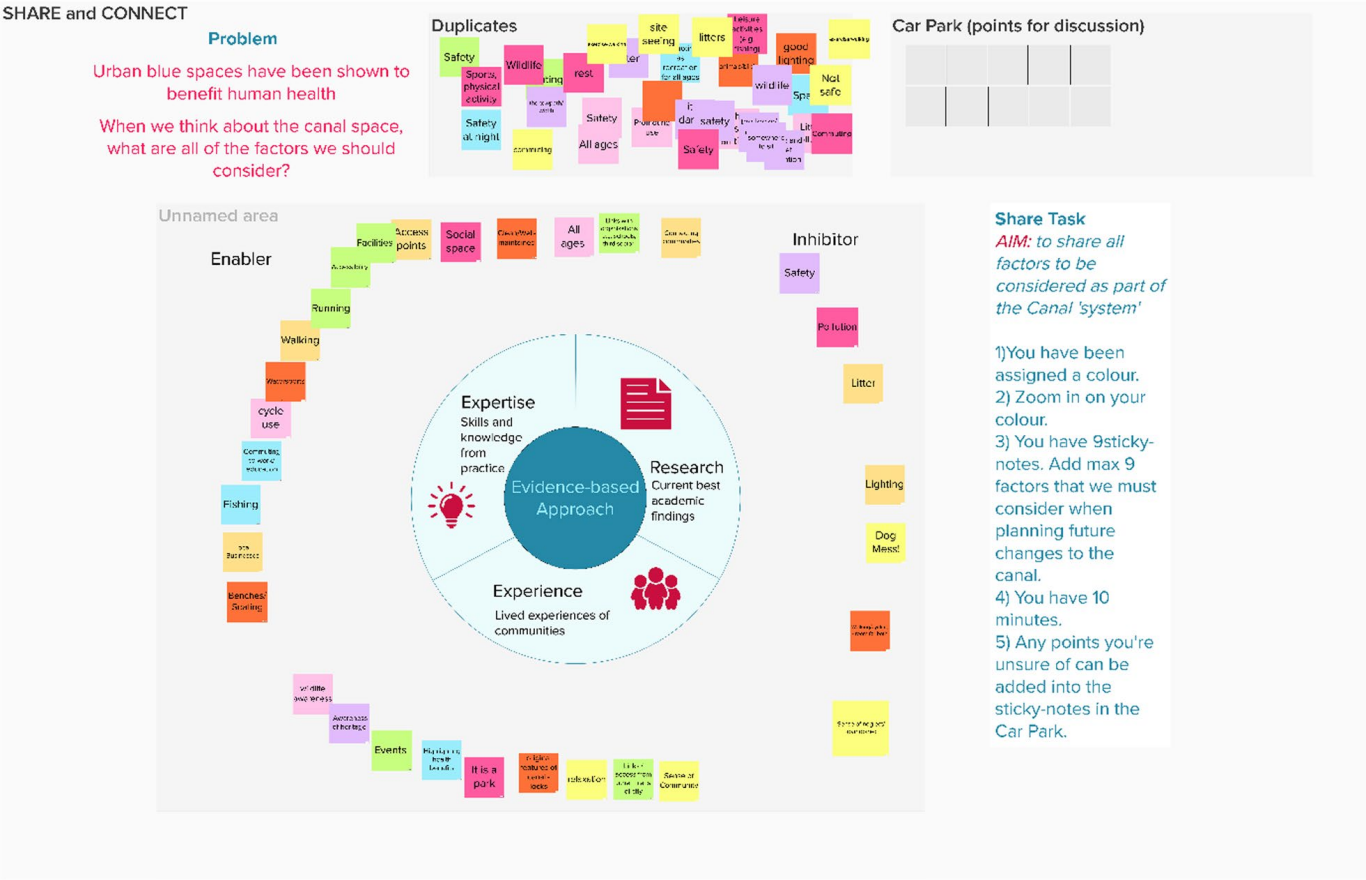
‘Share’ step conducted on Mural

The facilitator then went through all the variables with the participants to identify duplicates. We then arranged variables into a circle, where one side focused on ‘enablers’. These variables had a positive sentiment. The other side listed ‘inhibitors’, variables with a negative sentiment that were potential barriers in the system (Fig. 3).

### Connect

This step requires participants to start thinking about the links between the variables to construct an inter-relationship digraph (IRD) [45–47]. Participants are guided to review the variables elicited through ‘Share’ and must think about how these are related to each other. They can then connect these variables with a series of lines, creating the IRD. Through this process, participants are encouraged to consider all possible direct interactions between the variables. This process acknowledges the collective expertise of the participants, allowing them to share their perspectives and discuss links between the variables.

Infographics and other visual representations of fundamental ideas are used to interpret data, convey complex information and aid decision-making processes [58]. Representing systems through visual models is at the core of most systems approaches for managing complex issues. Systems studies employ standardised systems diagrams as they allow for a neat visualisation and simplification of how ideas and processes are interconnected.

### Blue space example

**Fig. 4:**
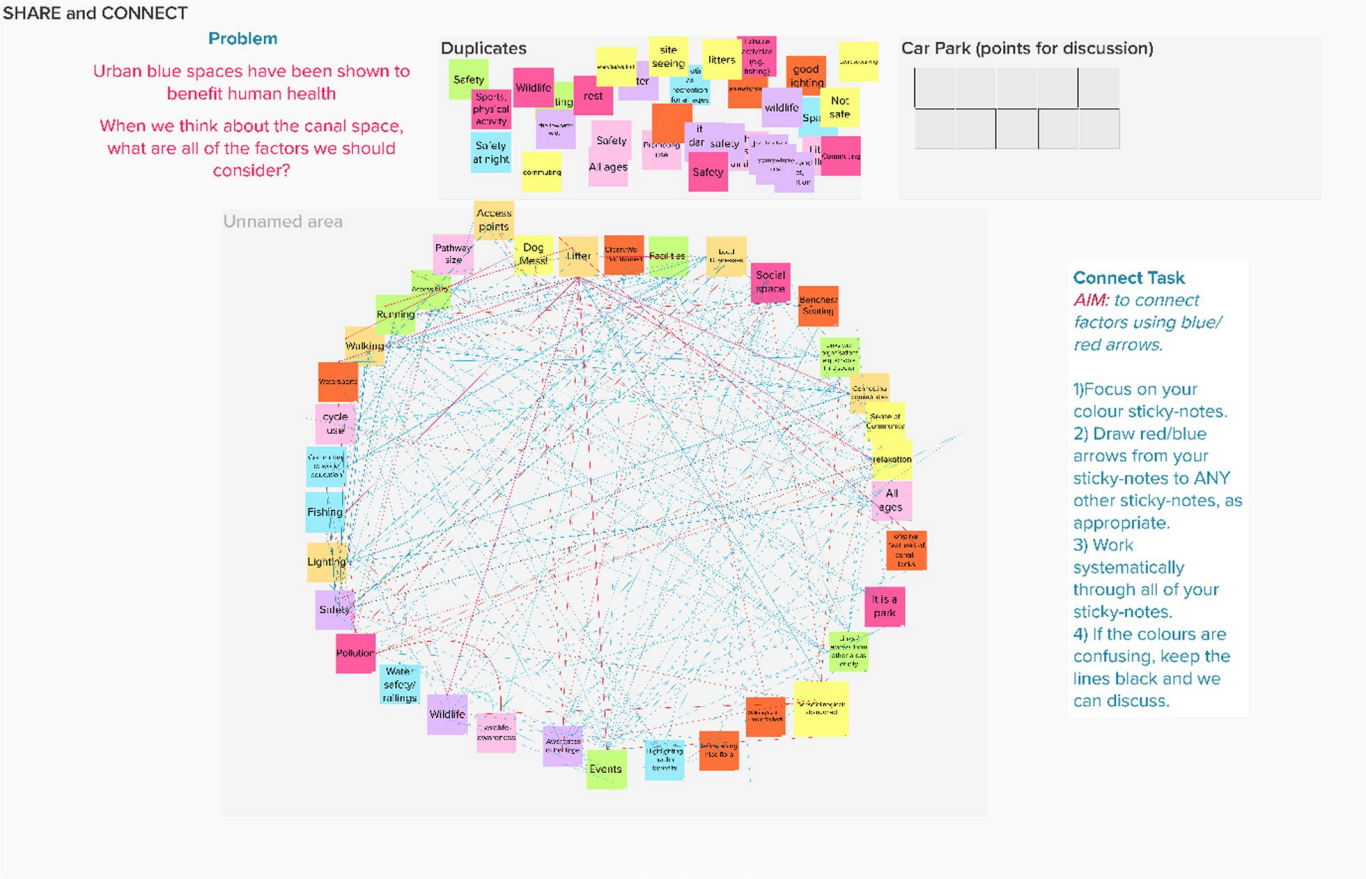
IRD showing connections between variables produced in Mural. Blue lines indicate positive polarity and red lines indicate negative polarity

In our project, participants developed a complex IRD, which showed the variables involved in the system of Canals and Health and the connections between them all (Fig. 4). Following the Share step, participants were invited to think through all of the possible connections that exist between the variables. All participants had access to the interactive whiteboard space and were invited to draw lines between the variables. Red and Blue lines were used to differentiate between polarity of the connections lines (see below for more on polarity). This IRD was achieved in one online session through discussion and participants having the opportunity to justify their choices. In total there were 26 variables connected through 208 connecting lines.

### Operationalise

Following the completion of an IRD, researchers must dissect the connections and look to refine and Operationalise the model to be operational for decision-making [47]. It is necessary to work through the data generated and synthesise the finding to operationalise the model, using a software package like Vensim, Stella, KUMU, STICKE, among others. Through this process, the researcher can look for feedback loops, shown through causal loop diagrams (CLDs). CLDs are an essential tool for diagramming the feedback structures of systems, used to visualise and communicate causal relationships. They represent qualitative mental models of their creators. Simulation models like stock and flow diagrams and agent-based models can be developed from a CLD.

**Fig. 5:**
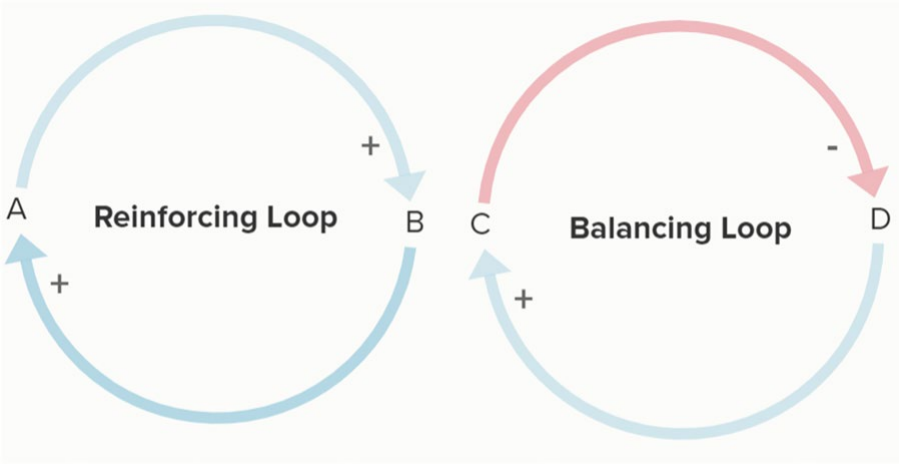
Types of feedback loops within CLDs

The components and language used within CLDs must be understood in order to make sense of them [48]. Causality refers to the relationship between two variables. The direction in which these relationships occur is indicated by polarity, which can be either positive or negative. Polarity is positive if changes in A and B are in the same direction i.e. both increase or both decrease. There is negative polarity when the changes in C and D are in the opposite direction i.e. C increases but D decreases and vice versa (Fig. 5).

The central components of CLDs are feedback loops. Feedback loops exist where changes in the system act as a catalyst through over variables to either reinforce or balance the initial change (Fig. 5). Reinforcing feedback loops signify amplifying effects. These are sometimes referred to as having an ‘avalanche’ effect. Walking through the reinforcing feedback loop, if A is assumed to increase, it results in an increase in B, which in turn causes further increase in A compared to the initial change, creating virtuous cycle (the loop between A and B in Fig. 5). If A is assumed to decrease, the loop creates a or vicious cycle. On the other hand, balancing Loops signify a dampening effect in the system. Within the balancing loop in Fig. 5, an initial increase in variable C in return results in a decrease in C (and vice versa). Loops can be analysed by exploring whether the narrative of the loop creates reinforcing or balancing behaviour. Loops can be given titles to indicate which way the reader should follow the loop, allowing for easier interpretation of the diagrams. Further information on this process has been well documented (see [10] chapter on Participatory Systems Mapping).

### Blue space example

Researchers worked through the IRD to unpick the loops surrounding how urban blue spaces affect health and all of the different variables that must be considered when thinking about changes to urban blue spaces. As technology develops, automated tools are emerging that convert the online post-it notes with lines between them into causal loop diagrams (e.g. Sticky Studio). This software was unavailable at the time of our project, so the Operationalise step was completed manually; we systematically looked at each connection and remapped it in an organised way in Kumu. Although time-consuming, manually conducting this step allowed us narratively work through the connections, which allowed us to become more familiar with the data and ultimately understand the system better.

CLDs can be used as seed models for quantitative simulation models, however, such methods were deemed unnecessary to this project. Although the complete CLD is not included in this paper, a future publication will present it in detail and focus on the findings and recommendations derived from it.

### Validate

Guba and Lincoln’s framework for ensuring trustworthiness and rigour in qualitative primary data collection can be used, which assesses research on four criteria: credibility, transferability, dependability, and confirmability [49]. Examples of ways to assess these dimensions of trustworthiness are presented below.

### Blue space example

We used a series of techniques to achieve the four dimensions of trustworthiness in our study. We used triangulation, where we used multiple sources of data, including quantitative evidence from the literature on the relationships between blue space and health, the output from the co-creation sessions and from follow up interviews with additional participants, to cross-check and validate findings. Comparing our map to existing literature allowed us to assess its generalizability and external validity [49].

We used member checking where we broke down the CLD into smaller loops and invited participants to narratively work through these to test clarity, ensure an accurate representation of relationships and check for missing information [25]. Using smaller loops is essential to ensure participants can understand and follow the details, otherwise using a full CLD at once would be overwhelming for participants which reduces the output of validation step. Including member checking ensured that the whole process of creating the CLD was participatory, from its initial conception to the refinement. In addition to member checking with participants, we also presented the operationalised map to stakeholders as we continued working with them including government officials, third sector organisations and at various online events. This step allowed us to gain feedback on the map and authenticate that the visualisation accurately depicted the consensus of how the canals work to influence health as a system.

We considered the dependability of findings, through conducting peer debriefings where we invited other researchers to review the research process and provide feedback. It was also crucial for us to maintain impartiality and be continually reflexive throughout the research process [49]. Following each encounter, the main researcher would write reflective notes on the session and discuss these with colleagues shortly after.

### Evolve

The development, refinement and analysis of the CLD expose key pathways and leverage points in the system, where potential strategies can be introduced to influence how it operates. These leverage points signal areas where we can intervene in a system to bring about positive change. Such engagement can stimulate new forms of action to propose ambitious changes in complex situations [50]. Engaging with CLD also allows us to anticipate and assess potential unintended consequences of any future changes. Often when interventions in a system fail, policy resistance mechanisms were not taken into account or not anticipated. The CLD may also highlight gaps in our current understanding and could be used to identify areas of future research.

### Blue space example

The development, refinement and analysis of the CLD for the blue space and health system enabled the identification of key pathways and leverage points where potential interventions could be introduced to influence the system’s functioning. We identified that the data generated from DISCOVER aligned with the four key mechanisms that we understand to link blue space and health; physical activity, social interaction, stress reduction and the environmental conditions [59]. Actions which addressed these mechanisms were likely to bring about positive change as they aligned with existing evidence but also reflected the world views of participants.

### Respond

The CLD and the action plan, developed through an analysis of the pathways, leverage points, and resulting scenarios, can inform innovations and policy change [5]. If policy changes are made, based on the CLD, it will be necessary to revisit the CLD to monitor any changes in proposed programs or interventions over time. Policymakers and urban planners must work flexibly to address the dynamic and adaptive properties of health systems, and engaging with CLDs can be a step towards achieving this. Furthermore, scaling such policies so that they can be shared as examples of good practice globally is an exciting prospect, which will also require an understanding of contexts and complexity [60].

The Respond step allows us to plan for significant and sustained change where interventions at many levels of the system work together synergistically. Participants propose their ideas to formulate an action plan for moving forward [61]. To develop the action plan, one can explore potential actions that are both highly scenario-dependent, as well as those that appear robust in their impact when considered within most scenarios [62]. At this stage, existing policy stress testing methodologies can provide valuable insights into potential vulnerabilities and weak points of interventions, helping reinforce them and make them more robust and resilient [63].

### Blue space example

The final Respond step was actioned through a final two-hour online workshop. Key decision-makers working around blue spaces and health were invited to participate via email. We worked in partnership with the Hydro Nation Chair Research & Innovation Programme to integrate their expertise in policy and reduce siloed working. In particular, we worked with their ‘Innovation: Place’ representative, who aims to develop and enhance the links between the Scottish water sector’s drive to net zero and the creation of greener, fairer, flourishing places. This partnership allowed us to improve the communication and dissemination of our research, helping us reach a wider audience with our policy recommendations. We drew on this partnership to attract key decision makers to our final co-creation session, and having them as a ‘gatekeeper’ encouraged other people to accept the invitation to participate [64]. The aim of the workshop was “To co-create a Blueprint for Improving Health and Health Inequality using Blue Spaces”. The agenda for our session is detailed in Table 2.

**Table 2:** Agenda for two-hour ‘Respond’ co-creation session

Time (minutes)	Task	Description
10	Welcome and Introduction	Welcome participants. Introduce the research on blue spaces and introduce the delivery team
15	Activity 1	What existing efforts have been made to improve health using blue spaces?
10	Introduction to systems thinking	Thinking through the consequences of actions
15	Activity 2	What other actions could be taken to improve health using blue spaces?
10	Break	
15	Activity 3	Prioritise actions through voting
30	Activity 4	Breakout room task to develop actions
15	Next Steps and Close	Outline next steps and invite feedback

Following this final session, actions were thematically analysed and grouped into sets of actionable recommendations. These centres around the four mechanisms we know to exist between blue space and health: physical activity, socialisation, mental health and wellbeing, and the environment. There are 12 actions in total, which encompass and synthesise the findings from our DISCOVER process. In a future article, we will provide further details of the output from the DISCOVER process.

If policy changes are made, based on our recommendations, it will be necessary to revisit the CLD to monitor any perturbations in proposed programs or interventions over time. Policymakers and urban planners must work flexibly to address the dynamic and adaptive properties of health systems, and engaging with CLDs can be a step towards achieving this. Following this research, we will be interested in further understanding how our findings can be scaled up sustainably across Scotland.

## Discussion

This paper has introduced the eight steps of the DISCOVER framework illustrated with a case study of how it has been used to respond to a complex problem. DISCOVER is an end-to-end planning and governance framework that offers a practical, systematic approach to address complex problems using a systems-based approach through co-creation.

### Building on existing systems methods

Group model building (GMB), which emerged as a way to extract mental models from stakeholders, allowing them to provide their perspective on a problem and propose solutions [11], is central in DISCOVER. Variations on the method exist, including ‘mediated modeling’ [30], ‘participatory system dynamics’ [47], participatory systems mapping [4] and ‘community based system dynamics’ [65], yet all are largely based on GMB principles. Perspectives and collective understanding shift throughout the modeling process as people share their thoughts, discuss core factors and collectively analyse the problem, with the aim of overcoming some of the problems intrinsic to linear thinking and nonparticipatory, siloed decision-making.

The DISCOVER framework complements GMB methods. The Share, Connect and Operationalise steps are effectively steps in a GMB project. Yet, DISCOVER augments the GMB process by having initial (Database and Identify) and finalising (Validate, Evolve, Respond) steps, to improve the planning and governing of the process, and respond to the output. Particularly, the first two steps systematically support with how to sample for GMB to ensure there is diversity in participants following a transparent process. This careful planning should ultimately improve the validity of the model. How to select participants for GMB tends to be vague in previous literature [10, 28, 30]. The Database step develops an exhaustive list of all possible people or organisations who have a vested interest in the topic and the social network analysis helps highlight ‘influencers’ within a network. The Identify checklist then incorporates different methods of sampling to create a transparent framework for sampling participants, something which is largely absent in the literature to date [10, 11, 28–30].

### Working virtually

DISCOVER was developed as an online process. Traditional GMB has taken place in person. However, during the COVID-19 pandemic, many researchers had no option but to move their GMB process online. This led to strides in innovative ways to conduct GMB workshops [66]. In our own research project, the move to online research required intensive upskilling as we had to navigate the best methods of online engagement. Also, all of our stakeholders had to move their own work online and adapt to alternative ways of working, which ultimately slowed down the stakeholder engagement process. Online research potentially ended up leading to higher engagement from some key stakeholders who may not have been able to commit to in person sessions. The exponential improvements that took place in digital engagement tools and online meeting platforms also meant that the data collection and analysis process was probably more efficient than if we had conducted GMB in person, although this is impossible to compare. However, there were some drawbacks with online data collection as it was more difficult to reach community groups. At the time, community groups were on the front line reacting to the COVID-19 situation and did not all have the capacity to engage. We therefore waited until restrictions eased and met some community group representatives in person to ensure we did not miss their input, which ultimately prolonged data collection.

### Strengths and limitations

A key strength is that DISCOVER is pragmatic and usable, guiding the user through the entire process in a systematic and transparent way. It ensures rigour and transparency in the research process as reporting can follow the same format as the framework, thus guiding the reader through the process. A further strength of the framework is that it is not entirely prescriptive; there are suggested methods for each step, but a number of alternative tools and methods could potentially be adopted should they still meet the objectives and aim. Other policy areas might be better suited to different analytical and collaborative methods, thus creating space for the different needs of researchers.

The framework is not without its limitations. We acknowledge the “fallacy of misplaced concreteness”, the issue of treating abstract concepts as if they were concrete physical objects, which can result in misunderstandings or oversimplifications [67]. This fallacy occurs when we mistake the abstract representation of something for the thing itself, rather than recognising it as a symbol or mental construct. It can lead to reification, the process of treating an abstract idea as if it were a real thing, and can be a barrier to effective communication and critical thinking. For example, reducing ‘sense of community’ to one variable may run the risk of oversimplifying the concept. To avoid this it is essential to maintain a reflective and critical approach, continually questioning the assumptions and simplifications made throughout the DISCOVER process. The Identify step also reduces the risk of reification by ensuring a diverse group of stakeholders are included in the process. We recognise that the model we created is a context-specific snapshot representation of the world and therefore should be considered as such.

## Conclusions

We have developed the DISCOVER framework for planning and governing system thinking co-creation. The framework increases rigour and transparency in system approaches to tackling complex issues going from planning to action. We invite others to use DISCOVER for their projects. The framework could be incorporated into research proposals and protocols for future projects. Finally, we welcome any feedback and refinement of the framework in the future.

### Supplementary Information

**Additional file 1: **Example’ Identify’ Checklist for stratified purposeful sampling.

## Data Availability

All data generated or analysed during this study are included in this published article.

## References

[1] Ison R. Systems Practice: How to Act. second. Milton Keynes: The Open University; 2017. 354 p.

[2] Reason P, Bradbury H, Ison R. Systems Thinking and Practice for Action Research. In: The SAGE Handbook of Action Research. 2014.

[3] Reynolds M, Holwell S. Systems approaches to making change: a practical guide. Springer; 2020.

[4] Penn AS, Barbrook-Johnson P. Participatory Systems Mapping: a practical guide. 2019. Available from: https://www.cecan.ac.uk/wp-content/uploads/2020/09/PSM-Workshop-method.pdf.

[5] Ison R (2017). Systems practice: how to act.

[6] Atkinson JAM, Wells R, Page A, Dominello A, Haines M, Wilson A. Applications of system dynamics modelling to support health policy. Public Health Res Pract. 2015; 10.17061/phrp2531531 26243490

[7] Peters DH (2014). The application of systems thinking in health: why use systems thinking?. Health Res Policy Syst.

[8] Bishai D, Paina L, Li Q, Peters DH, Hyder AA (2014). Advancing the application of systems thinking in health: why cure crowds out prevention. Health Res Policy Syst.

[9] Adam T. Advancing the application of systems thinking in health. Health Research Policy and Systems. 2014. 10.1186/1478-4505-12-50 PMC4245197 25160646

[10] Barbrook-Johnson P, Penn AS. Systems Mapping: how to build and use causal models of systems. Cham: Springer International Publishing; 2022. 10.1007/978-3-031-01919-7.

[11] Vennix JAM (1996). Group model building: facilitating team learning using system dynamics.

[12] Baum F. The New Public Health. Oxford University Press; 2016. 720 p.

[13] Namin AT, Jalali MS, Vahdat V, Bedair HS, O’Connor MI, Kamarthi S (2019). Adoption of new medical technologies: the case of customised individually made knee implants. Value Health.

[14] Gaveikaite V, Grundstrom C, Lourida K, Winter S, Priori R, Chouvarda I, et al. Developing a strategic understanding of telehealth service adoption for COPD care management: a causal loop analysis of healthcare professionals. PLoS ONE. 2020; 10.1371/journal.pone.0229619 PMC7058286 32134958

[15] Bar-Yam Y (2006). Improving the effectiveness of health care and public health: a multiscale complex systems analysis. Am J Public Health.

[16] Jalali MS, Rahmandad H, Bullock SL, Lee-Kwan SH, Gittelsohn J, Ammerman A (2019). Dynamics of intervention adoption, implementation, and maintenance inside organisations: the case of an obesity prevention initiative. Soc Sci Med.

[17] Stringfellow EJ, Lim TY, Humphreys K, DiGennaro C, Stafford C, Beaulieu E (2022). Reducing opioid use disorder and overdose deaths in the United States: a dynamic modeling analysis. Sci Adv.

[18] Lim TY, Stringfellow EJ, Stafford CA, DiGennaro C, Homer JB, Wakeland W (2022). Modeling the evolution of the US opioid crisis for national policy development. Proc Natl Acad Sci.

[19] Buck C, Loyen A, Foraita R, Van Cauwenberg J, De Craemer M, Mac DC (2019). Factors influencing sedentary behaviour: a system based analysis using Bayesian networks within DEDIPAC. PLoS ONE.

[20] Chastin SFM, De Craemer M, Lien N, Bernaards C, Buck C, Oppert JM (2016). The SOS-framework (Systems of Sedentary behaviours): an international transdisciplinary consensus framework for the study of determinants, research priorities and policy on sedentary behaviour across the life course: a DEDIPAC-study. Int J Behav Nutr Phys Act.

[21] Friel S, Hattersley L, Ford L, O’Rourke K. Addressing inequities in healthy eating. Health Promot Int. 2015; 10.1093/heapro/dav073 26420812

[22] Levy DT, Mabry PL, Wang YC, Gortmaker S, Huang TTK, Marsh T (2011). Simulation models of obesity: a review of the literature and implications for research and policy. Obes Rev.

[23] McGlashan J, Johnstone M, Creighton D, De La Haye K, Allender S (2016). Quantifying a systems map: network analysis of a childhood obesity causal loop diagram. PLoS ONE.

[24] Vandenbroeck P, Goossens J, Clemens M. Tackling obesities: future choices—building the obesity system map. Foresight. 2007.

[25] Rwashana AS, Nakubulwa S, Nakakeeto-Kijjambu M, Adam T (2014). Advancing the application of systems thinking in health: understanding the dynamics of neonatal mortality in Uganda. Health Res Policy Syst.

[26] Smith N, Georgiou M, King AC, Tieges Z, Webb S, Chastin S (2021). Urban blue spaces and human health: a systematic review and meta-analysis of quantitative studies. Cities.

[27] Dick B, Sankaran S, Shaw K, Kelly J, Soar J, Davies A (2015). Value co-creation with stakeholders using action research as a meta-methodology in a funded research project. Proj Manag J.

[28] Hovmand PS, Etiënne, Rouwette AJA, Andersen DF, Richardson GP, Kraus A. Scriptapedia 4.0.6. 2013.

[29] Scott R. Group Model Building [Internet]. Singapore: Springer; 2018. (SpringerBriefs in Operations Research). 10.1007/978-981-10-8959-6.

[30] van den Belt M, Dietz T. Mediated Modeling: A System Dynamics Approach to Environmental Consensus Building [Internet]. Washington, D. C., UNITED STATES: Island Press; 2004. Available from: http://ebookcentral.proquest.com/lib/gcal/detail.action?docID=3317433.

[31] Higgins JPT, Thomas J, Chandler J, Cumpston M, Li T, Page M, et al., editors. Cochrane Handbook for Systematic Reviews of Interventions. version 6.3 (updated February 2022). 2022 [cited 2023 Aug 16]. Available from: www.training.cochrane.org/handbook.

[32] Sage Publications Inc. 2023 [cited 2023 Apr 14]. The SAGE Encyclopedia of Qualitative Research Methods. Available from: https://us.sagepub.com/en-us/nam/the-sage-encyclopedia-of-qualitative-research-methods/book229805.

[33] Sage Publications Inc. 2023 [cited 2023 Apr 14]. The SAGE Handbook of Quantitative Methodology for the Social Sciences. Available from: https://us.sagepub.com/en-us/nam/the-sage-handbook-of-quantitative-methodology-for-the-social-sciences/book226672.

[34] Palinkas LA, Horwitz SM, Green CA, Wisdom JP, Duan N, Hoagwood K. Purposeful sampling for qualitative data collection and analysis in mixed method implementation research. Adm Policy Ment Health Ment Health Serv Res. 2015. 10.1007/s10488-013-0528-y PMC4012002 24193818

[35] Schütz F, Heidingsfelder ML, Schraudner M. Co-shaping the future in quadruple helix innovation systems: uncovering public preferences toward participatory research and innovation. She Ji. 2019;

[36] Fast Track Impact. [cited 2023 Apr 24]. The Research Impact Handbook (2nd Edition). Available from: https://www.fasttrackimpact.com/product-page/the-research-impact-handbook-2nd-edition-1.

[37] Maantay J, Maroko A (2015). ‘At-risk’ places: inequities in the distribution of environmental stressors and prescription rates of mental health medications in Glasgow, Scotland. Environ Res Lett.

[38] Baka A, Mabon L (2022). Assessing equality in neighbourhood availability of quality greenspace in Glasgow, Scotland, United Kingdom. Landsc Res.

[39] Tieges Z, Georgiou M, Smith N, Morison G, Chastin S (2021). Investigating the association between regeneration of urban blue spaces and risk of incident chronic health conditions stratified by neighbourhood deprivation: a population-based retrospective study, 2000–2018. Int J Hyg Environ Health.

[40] Georgiou M, Tieges Z, Morison G, Smith N, Chastin S. Does living near blue space modify the impact of socio-economic deprivation on mental health in urban areas? A population-based retrospective study. Sci Rep Nat Publ Group. 2022. 10.1038/s41598-022-17089-z PMC9338232 35906285

[41] Smith N, Foley R, Georgiou M, Tieges Z, Chastin S (2022). Urban blue spaces as therapeutic landscapes: “a slice of nature in the city”. Int J Environ Res Public Health.

[42] Creswell JW, Creswell JD. Research design: qualitative, quantitative, and mixed methods approaches. Fifth. SAGE Publications, Inc; 2018.

[43] Chiesi AM. Network Analysis. In: Wright JD, editor. International encyclopedia of the social & behavioral sciences (Second Edition). Oxford: Elsevier; 2015. p. 518–23. Available from: https://www.sciencedirect.com/science/article/pii/B9780080970868730558.

[44] Scott J. Social Network Analysis. SAGE Publications Ltd; 2017. Available from: https://methods.sagepub.com/book/social-network-analysis-4e.

[45] de Pinho H. Participant guidelines systems tools for complex health systems: a guide to creating causal loop diagrams. New York City; 2015.

[46] Lembani M, De Pinho H, Delobelle P, Zarowsky C, Mathole T, Ager A (2018). Understanding key drivers of performance in the provision of maternal health services in eastern cape, South Africa: a systems analysis using group model building. BMC Health Serv Res.

[47] Lembani M, de Pinho H, Delobelle P, Zarowsky C, Mathole T, Ager A. A guide for participatory systems analysis using a group model building approach. Guide Particip Syst Anal Using Group Model Build Approach. 2020;

[48] Hummelbrunner R, Williams B. Systems concepts in action: a practitioner’s toolkit. Stanford: Stanford University Press; 2010. 336 p. Available from: http://www.sup.org/books/title/?id=18331.

[49] Guba EG, Lincoln YS. Fourth Generation Evaluation [Internet]. SAGE Publications; 1989. Available from: https://books.google.co.uk/books?id=k_zxEUst46UC.

[50] Shaw DP. Changing conversations in organisations. Changing Conversations in Organisations. 2003.

[51] Hovmand PS, Andersen DF, Rouwette E, Richardson GP, Rux K, Calhoun A (2012). Group model-building ‘scripts’ as a collaborative planning tool. Syst Res Behav Sci.

[52] Greenhalgh T, Jackson C, Shaw S, Janamian T (2016). Achieving research impact through co-creation in community-based health services: literature review and case study. Milbank Q.

[53] Friedman SR, Neaigus A, Jose B, Curtis R, Des JD (1998). Networks and HIV risk: an introduction to social network analysis for harm reductionists. Int J Drug Policy.

[54] Hanneman RA, Riddle M. Introduction to social network methods. Riverside CA Univ Calif Riverside -Line Textb. 2005;

[55] DiMaggio P, Garip F (2012). Network effects and social inequality. Annu Rev Sociol.

[56] Leask CF, Sandlund M, Skelton DA, Altenburg TM, Cardon G, Chinapaw MJM (2019). Framework, principles and recommendations for utilising participatory methodologies in the co-creation and evaluation of public health interventions. Res Involv Engagem.

[57] Andersen DF, Richardson GP (1997). Scripts for group model building. Syst Dyn Rev.

[58] Otten JJ, Cheng K, Drewnowski A (2015). Infographics and public policy: using data visualisation to convey complex information. Health Aff (Millwood).

[59] Georgiou M, Morison G, Smith N, Tieges Z, Chastin S (2021). Mechanisms of impact of blue spaces on human health: a systematic literature review and meta-analysis. Int J Environ Res Public Health.

[60] Paina L, Peters DH (2012). Understanding pathways for scaling up health services through the lens of complex adaptive systems. Health Policy Plan.

[61] Roberts N. Wicked problems and network approaches to resolution. Int Public Manag Rev. 2000.

[62] Chipperfield T, O’Brien R, Bolderson T, Eidinow E, Shafner L. Qualitative Modelling of Policy Options. 2007.

[63] European Parliament. Directorate General for Parliamentary Research Services. How to stress-test EU policies: building a more resilient Europe for tomorrow. LU: Publications Office; 2022. 10.2861/301781.

[64] McFadyen J, Rankin J (2016). The role of gatekeepers in research: learning from reflexivity and reflection. GSTF J Nurs Health Care.

[65] Hovmand PS (2014). Community based system dynamics.

[66] Wilkerson B, Aguiar A, Gkini C, Czermainskide Oliveira I, Lunde Trellevik LK, Kopainsky B (2020). Reflections on adapting group model building scripts into online workshops. Syst Dyn Rev.

[67] Whitehead AN, Griffin DR (1978). Process and reality: an essay in cosmology.

