# Supplementary material for: Planning, implementing and governing systems-based co-creation: the DISCOVER framework
Source: Health Res Policy Syst. 2024 Jan 8;22:6. doi: 10.1186/s12961-023-01076-5 (PMC10773095; doi:10.1186/s12961-023-01076-5)
Supplement: Supplementary file 1 — Additional file 1: Example’ Identify’ Checklist for stratified purposeful sampling. [file 12961_2023_1076_MOESM1_ESM.docx]

**Appendices**

**Appendix 1**Example' Identify' Checklist for stratified purposeful sampling. Here, the user can add organisations' names to each box. By the end of the sampling, there should be at least one organisation attributable to each item on the checklist.

|  | 1 | 2 | 3 | 4 | 5 | 6 | 7 | 8 | 9 | 10 | 11 | 12 | Total |
| --- | --- | --- | --- | --- | --- | --- | --- | --- | --- | --- | --- | --- | --- |
| Network Analyses |  |  |  |  |  |  |  |  |  |  |  |  |  |
| Leader within the System (Eigenvector) |  |  |  |  |  |  |  |  |  |  |  |  |  |
| Local Hubs (Degree Centrality) |  |  |  |  |  |  |  |  |  |  |  |  |  |
| Spreaders/ High Visibility into what is happening (Closeness Centrality) |  |  |  |  |  |  |  |  |  |  |  |  |  |
| Bridges in the network (Betweenness Centrality) |  |  |  |  |  |  |  |  |  |  |  |  |  |
| **Quadruple Helix Model** | | | | | | | | | | | | | |
| - Academic |  |  |  |  |  |  |  |  |  |  |  |  |  |
| - Government |  |  |  |  |  |  |  |  |  |  |  |  |  |
| - Business |  |  |  |  |  |  |  |  |  |  |  |  |  |
| - Civil Society |  |  |  |  |  |  |  |  |  |  |  |  |  |
| **Sector of Expertise** | | | | | | | | | | | | | |
| - Health |  |  |  |  |  |  |  |  |  |  |  |  |  |
| - Regeneration (past and present) |  |  |  |  |  |  |  |  |  |  |  |  |  |
| - Community Use of the Space |  |  |  |  |  |  |  |  |  |  |  |  |  |
| - Environment |  |  |  |  |  |  |  |  |  |  |  |  |  |
| - Policy / Regulations |  |  |  |  |  |  |  |  |  |  |  |  |  |

**Appendix 2
Variable Elicitation Script for Blue Space and Health Workshop**

Adapted from Hovman et al. (2015) Script detailed here: <https://en.wikibooks.org/wiki/Scriptapedia/Variable_Elicitation>

**Purpose:** Identifying variables for building IRDs and CLDs

**Time:** 30 minutes

**Materials needed:**

1. Mural template
2. Video conferencing platform
3. Reliable internet connection

**Outputs:** List of variables that exist between urban waterways and health

**Script:**

"Our goal today is to identify the variables that exist between urban waterways and health.

Firstly, let's define the problem. We know that there are links between urban blue spaces and health, but we do not fully understand these links. If we want to maximise the health benefits of urban blues spaces, we must work out what the links are. What do you think are the factors that link these two things? Please add sticky notes to the mural with the factors that you think link urban blue spaces and health. Let's take 10 minutes to do this. I will set the timer at the top of the screen. If you are unsure about how to word the factor, take a note yourself and we can discuss it together after the 10minutes is up.

*Set 10minute timer*

Thank you, it looks like we have lots of variables to talk through. There is likely to be some duplication, so let's take a look and try to group the factors. Were there any factors that you found difficult to summarise in a sticky note? Let's talk through these and we can try to give them succinct labels together. Are there any variables which you cannot see here? Is there anything we are missing?

Let's discuss the variables we have identified. Are these all clear to all stakeholders? Can they be further grouped? Do we understand the story behind the variables? Are all ideas summarised? Has everyone had a chance to contribute?

Our next lab will look to connect these variables and start to understand the relationships between them.

Thank you for your contribution today. This is an exciting project and I look forward to meeting with you again soon to continue exploring this relationship between urban blue spaces and better health for the people of Glasgow and beyond!"
